# Hypoglycaemic events in patients with type 2 diabetes in the United Kingdom: associations with patient-reported outcomes and self-reported HbA1c

**DOI:** 10.1186/1472-6823-13-59

**Published:** 2013-12-19

**Authors:** Beth D Mitchell, Jeffrey Vietri, Anthony Zagar, Bradley Curtis, Matthew Reaney

**Affiliations:** 1 Eli Lilly & Company, Indianapolis, IN, USA; 2 Kantar Health, Health Outcomes Practice, Milan, MI 20121, Italy; 3 Eli Lilly & Company, Windlesham, Surrey, UK

**Keywords:** Hypoglycaemia, Hypoglycaemic events, Health related quality of life, Hypoglycaemia fear, Treatment satisfaction

## Abstract

**Background:**

One possible barrier to effective diabetes self-management is hypoglycaemia associated with diabetes medication. The current study was conducted to characterize hypoglycaemic events among UK patients with type 2 diabetes (T2D) treated with antihyperglycaemic medications, and assess the relationship between experience of hypoglycaemic events and health outcomes, including glycaemic control, health-related quality of life, impairment to work and non-work activities, treatment satisfaction, adherence to treatment, fear of hypoglycaemia, and healthcare resource use.

**Methods:**

An online survey of 1,329 T2D patients in UK drawn from an opt-in survey panel was conducted in February of 2012 with monthly follow-up questionnaires for five months. Measures included self-reported HbA1c, EQ-5D, Work Productivity and Activity Impairment questionnaire, Diabetes Medication Satisfaction Tool, Morisky medication adherence scale, the Hypoglycaemia Fear Survey (revised), and self-reported healthcare resource use. Comparisons were conducted using t-tests and chi-square tests for continuous and categorical variables, respectively.

**Results:**

Baseline comparisons showed that worse HbA1c, greater diabetes-related healthcare resource use, greater fear of hypoglycaemia, and impaired health outcomes were associated with experience of hypoglycaemia in the four weeks prior to baseline. Longitudinal results were similar in direction but differences on few measures were significant.

**Conclusions:**

In real-world UK T2D patients, hypoglycaemia is associated with worse self-reported glycaemic control, behaviours that contribute to worse glycaemic control, and impairment in patient-reported outcomes.

## Background

Numerous studies have demonstrated that intensive control of blood glucose reduces the development and progression of vascular complications in diabetes [1-7]. The balance of randomized trial evidence suggests intensive glucose control reduces risk for some cardiovascular disease outcomes [8], even while there are notable exceptions to this pattern [1,9,10].

However, even patients who are placed on intensive treatment often do not achieve adequate control, a result observed in both clinical trials and observational studies [11-14]. One potential reason for inadequate control is the experience or fear of hypoglycaemia, which can lead to non-adherence or non persistence to treatment, and other behaviours to raise blood glucose, such as increased consumption of carbohydrates, avoidance of exercise, and reducing the dose of antihyperglycaemic medications [15-18]. Further, physicians see hypoglycaemia as a limiting factor in how aggressively they can use therapies such as insulin (associated with hypoglycaemia) to treat type 2 diabetes (T2D) [19].

The relationship between hypoglycaemic events (HEs) and outcomes in patients with T2D has been investigated in several recent studies, including a cross-sectional survey of 2,074 patients with T2D in the United States (US) on oral antihyperglycaemic medication [20]. Those who reported symptoms of hypoglycaemia at least ‘some of the time’ on the hypoglycaemia items of the Diabetes Symptom Measure [21] were less likely to be at their treatment goal for glycosylated haemoglobin (HbA1c), as well as less likely to know their HbA1c.

Another large (n = 1,984) survey of US patients with T2D found worse self-reported health status as severity of HE increased from mild (no interruption of activities) through moderate, severe, and very severe (required medical attention) [22]. The same study found a relationship between frequency of HEs and increased worry about hypoglycaemia. Likewise, linking patient surveys with healthcare claims data demonstrates that patients with T2D treated with pharmacotherapy who experience HEs have higher medical costs, more fear of hypoglycaemia, and impaired health status relative to patients without HEs [23].

Further, a multi-country study in Asia (n = 2257) assessed the relationship between experience of symptoms of hypoglycaemia and HbA1c, health status, and worry about hypoglycaemia [24]. As in the studies above, hypoglycaemia symptoms were associated with worse health status and more worry about hypoglycaemia. This study also measured HbA1c, and an unadjusted analysis showed 0.24% *lower* average HbA1c among those with HE in the 6 months prior. Patient-perceived symptom severity was not associated with differences in HbA1c, though the pattern of means suggests more severe HE may be associated with higher HbA1c.

Despite these recent additions to the literature, gaps remain, especially within individual countries. It is important to understand how blood glucose levels, particularly the longer-term blood glucose levels reflected in HbA1c, are related to the experience of HEs, a relationship seldom assessed. Therefore, the objective of current study was to assess the link between HEs, HbA1c, patient-reported outcomes, and healthcare resource use among patients with T2D in the United Kingdom (UK).

## Methods

Potential respondents were identified through the 2011 5EU National Health and Wellness Survey (NHWS) and the diabetes chronic ailment panel of Light Speed Research in the UK. The 5EU NHWS is a proprietary survey of adults (≥18 years) conducted by Kantar Health in France, Germany, Italy, Spain, and UK, which collects data approximately every 18 months to provide timely patient-reported information on a broad range of health conditions. The UK portion of the 5EU NHWS survey includes 15,000 respondents representative of the UK population in terms of age and gender. The Light Speed chronic ailment panel consists of individuals who have previously indicated they suffer from diabetes. All panel participants were originally sourced from a general (i.e., not healthcare-specific) internet survey panel whose members are recruited through opt-in emails, co-registration with panel partners, e-newsletter campaigns, online banner placements, and both internal and external affiliate networks. All panelists explicitly consented to become panel members, registered through unique email addresses, and completed in-depth demographic registration profiles.

Panelists from the Light Speed chronic ailment group and respondents to the 5EU NHWS survey residing in the UK who indicated that they had diabetes were sent an invitation email with a link to the informed consent of the survey (n = 7,144). Those who gave consent (n = 3,224; 45%) were then screened for a physician diagnosis of T2D (n = 2,396), and current use of a prescription medicine for their T2D (n = 2,071). In order to ensure adequate numbers of insulin users, a quota of 300 respondents using insulin was included, which resulted in 295 T2D patients using only oral medication being excluded from the survey. The remaining patients (n = 1,776) were directed to the baseline questionnaire.

The study was designed as a longitudinal survey with six assessment points from February 2012-July 2012, each separated by four weeks. Respondents were informed that they would receive a small incentive for completion of each assessment point, either in the form of points that could be exchanged for merchandise or a monetary payment. Incentives increased from approximately £2.50 for the baseline survey to £12.75 for the final assessment to discourage attrition. Respondents were notified of the availability of the questionnaire via email at the opening of each assessment point and had one week to complete the questionnaire, and all those who completed the baseline were invited to participate in each follow-up. Up to four reminder emails were sent over the course of the week the survey was available. However, attrition over the duration of the study resulted in only 34% (n = 451) of the baseline sample reporting at the last follow-up, and only 12% (n = 155) of baseline respondents completed all follow-up waves. Therefore, the current study primarily focuses on cross-sectional analysis of baseline data.

The study protocol and questionnaire was approved by Essex IRB, a commercial IRB based in the United States.

### Measures

All measures were by self-report with the exception of geographic region, which was extracted from survey panel records. Demographic characteristics collected included age, gender, employment status, annual household income, and UK region. Height and weight were used to calculate body mass index (BMI), and respondents indicated whether they currently smoke cigarettes, as well as the number of days they exercised “vigorously” in the past 30. Respondents were also asked to indicate whether they had been diagnosed with various chronic conditions, which were used to calculate the Charlson comorbidity index (CCI) [25].

Questions regarding their experience with HEs included whether they had ever experienced a HE, the number of events they had experienced in the prior six months, and the number of HEs they had experienced in the prior four weeks. These were categorised by the level of assistance needed; self-managed, requiring assistance from someone else (not a healthcare professional), and requiring assistance from a medical professional.

Those who reported ever experiencing a HE answered questions characterizing the most recent event. These included how the event was detected (symptoms confirmed by a blood glucose reading, symptoms not confirmed with a blood glucose reading, and a low blood glucose reading in the absence of symptoms), the perceived trigger of the event, the assistance required, the circumstances (what they were doing at the time) and what actions they (or someone else) took in response to the HE.

Glycaemic control was assessed through the most recent self-reported HbA1c. Respondents could use either percentage or mmol/mol formats (here converted to percentage for reporting). Those who could not provide an HbA1c reading were presented with a series of ranges of HbA1c values and asked to indicate the range (and approximate date) of their most recent results. Because HbA1c is only typically assessed between one and four times per year in the UK, values provided at the first follow-up survey were used to supplement the values provided at baseline for those who did not provide a baseline value.

Multiple existing patient-reported outcome questionnaires were administered as part of the survey:

Health status was assessed using the EQ-5D questionnaire [26]; a widely used preference-based, generic (as opposed to disease-specific) questionnaire. The questionnaire consists of 2 parts – a population-weighted health state index score and a self-perceived current health score. Only the health state index score was used in this study. This score is calculated based on responses to 5 dimensions of health (mobility, self-care, usual activities, pain/discomfort and anxiety/depression) and then weighted using UK population weights to provide a single value on a scale from −0.594 to1 with higher scores indicating better health utility (negative values indicate a health state worse than dead).

The Work Productivity and Activity Impairment (WPAI) questionnaire [27] was used to measure impairment to work productivity and the activities of daily living. Four subscales (absenteeism, presenteeism, overall work impairment, and activity impairment) are generated in the form of percentages (0 to 100%), with higher values indicating greater impairment during the preceding seven days. All respondents completed the general health version, which queries work and activity impairment due to general health. Those reporting a hypoglycaemic event in the preceding seven days also completed the Specific Health Problem version to assess impairment due to hypoglycaemia.

Treatment satisfaction was measured using the Diabetes Medication Satisfaction Tool (DMSAT). The DMSAT [28] measures satisfaction with the patient’s diabetes medications regimen. The instrument consists of 16 items which create 4 subscales (3 items for wellbeing, 3 items for medical control, 5 items for lifestyle, and 5 items for convenience) and a total score. Responses are summed and converted to a score from 0 to 100 for each subscale and overall, with higher scores representing more satisfaction. For the purposes of this study, only the overall score was used.

Adherence to medication was measured using the Morisky medication adherence scale (MMAS) [29,30].The MMAS is a generic self-reported, medication-taking behaviour scale, consisting of four items which are summed to give a range of scores from high adherence (0) to low adherence (4). After discussion with the scale author, the scale was modified, creating one version assessing oral antidiabetic medication and a second assessing insulin (administered only to patients taking insulin).

The Hypoglycaemia Fear Survey, revised (HFS-II) was used to measure fear of hypoglycaemia [31]. This is a 33-item questionnaire with two subscales that measure 1) behaviours to avoid hypoglycaemia and its negative consequences and 2) worries about hypoglycaemia and its negative consequences. Responses are made on a 5-point Likert scale where 0 = Never and 4 = Always. The behaviour, worry, and total scores were all included in analyses. Behaviour subscale items include items that would not necessarily contribute to poorer glycaemic control (e.g., avoiding being alone) and five behaviors that would: eating large snacks, keeping blood glucose (BG) above 150, keeping BG higher than normal in social situations, keeping BG higher than normal during important activities, and reducing insulin dose. These items were included in the behavioral subscale and total scale scores, but also analyzed separately, dichotomized as never engaging in that behaviour versus engaging in the behaviour during the recall period. Though the standard recall period for the HFS-II is six months, the measure was modified to better suit the design of the current study to include a four-week recall period after discussion with the scale author.

In addition, respondents were asked to indicate the number of times they visited different types of healthcare providers in relation to their diabetes care during the four weeks prior as a measure of healthcare resource use. These included the physician who primarily manages their T2D (may be primary or secondary care), other physicians, and other providers (nurses, dieticians, podiatrists, etc.). These were combined with the primary physician visits to provide a count of the total provider visits. Another item queried the number of times the respondent was hospitalized in the preceding four weeks.

### Statistical analysis

The total sample was characterized using mean and standard deviation for continuous variables and frequency and percentages for categorical variables. These were followed with a comparison between the group of patients who reported having a hypoglycaemic event in the four weeks preceding baseline and the group who did not report an event, with comparisons planned for the outcomes listed above. For continuous variables, an independent groups t-statistic was used for the comparison (with a Welch-Satterthwaite approximation to the degrees of freedom when the homogeneity assumption violated). A chi-square test was used for categorical variables. P-values < 0.05 were considered statistically significant. No adjustment for multiplicity was made.

## Results

A total of 1,329 respondents completed the baseline survey; follow-up surveys were completed by 836, 759, 765, 511, and 451 respondents, respectively. Demographics and general health characteristics are presented in Table 1. Consistent with the T2D population, the sample was predominantly late middle age, male, and overweight. All the regions of the UK were represented, roughly in proportion to the general population. Clinical characteristics are presented in Table 2. Respondents on average reported diabetes duration of 9.6 years, and most were free of complications. The majority were using only oral antihyperglycaemic medication. Of those who provided a value for HbA1c (29.6%), the mean value was 7.3%. When considering both those who provided a value for HbA1c (n = 393) and those who could not provide a value but selected their HbA1c from a range (n = 495), 32.6% reported an HbA1c of 7.0% or below. An additional 33.2% did not provide either an exact or approximate HbA1c value.

**Table 1:** Demographics and general health characteristics of the sample (n = 1,329)

	**n/mean**	**%/SD**
Demographics		
Age	58.8	10.9
Female	534	40.2%
Employed	448	33.7%
Annual household Income		
	<30,000£	846	63.7%
	≥30,000£	330	24.8%
	Decline to answer	153	11.5%
General health			
BMI (continuous)	31.9	6.4	
BMI (categories)			
	Underweight	8	0.6%
	Normal weight	119	9.0%
	Overweight	362	27.2%
	Obese	683	51.4%
	Decline to answer	157	11.8%
Exercised*	599	45.1%	
Currently smoke	158	11.9%	
CCI	1.6	1.5	

*Note: Exercised indicates exercising “vigorously” at least once for 20 minutes during the preceding month. CCI: Charlson comorbidity index.

**Table 2:** Diabetes characteristics of sample (n = 1,329)

	**n/mean**	**%/SD**
Duration of diabetes (years)	9.6	7.0
HbA1c (among those providing a number; n = 393)	7.25%	1.5%
HbA1c in ranges		
Up to 6.5%	219	16.5%
6.6%-7.0%	214	16.1%
7.1%-7.5%	152	11.4%
7.6%-8.5%	146	11.0%
8.6%-9.5%	79	5.9%
9.6%-10.5%	44	3.3%
10.6% or above	34	2.6%
Unknown	441	33.2%
Medications		
Oral (OAD) only	971	73.1%
Non-insulin injectable (NII) only	16	1.2%
Insulin (IN) only	79	5.9%
OAD & NII	41	3.1%
OAD & IN	174	13.1%
NII & IN	7	0.5%
OAD, NII, & IN	41	3.1%
Complications		
Macular edema/diabetic retinopathy	117	8.8%
Kidney disease	51	3.8%
Foot or leg ulcer	60	4.5%
Neuropathic pain	220	16.6%
End organ damage	18	1.4%
None	989	74.4%

The prevalence of ≥1 HE within the 4-week period was 27.5% for the sample overall, higher among insulin users (n = 301) than those not using insulin (n = 1,028) (43.5% vs. 22.8%, p < 0.0001).

### Characteristics of most recent HE

Among those experiencing ≥1 HE, most (61.2%) had symptoms of hypoglycaemia with a low blood glucose reading, but 27.7% did not test blood glucose during the most recent HE. 5.5% were only aware of their most recent event because of a blood-glucose test, and 5.5% had a normal blood glucose reading during the event they considered to be hypoglycaemic in nature.

Among those experiencing symptoms during their most recent HE, the most common were trembling/shaking (55.3%), dizziness/light-headedness (53.6%), sweating (38.6%), weakness (34.1%) and uneasiness/ill feeling (32.6%). Most patients (89.8%) reported experiencing multiple symptoms.

Most HEs were attributed to lack of timely eating or inappropriate food choices. The most commonly endorsed reasons were delayed eating (29.6%), irregular or too few carbohydrates (24.8%) and skipped meals or snacks (20.4%). Too much exercise (15.6%) and stress (13.2%) were also commonly implicated triggers. 4.6% attributed the incident to a new antidiabetic medication, and 10% of insulin users experiencing a HE indicated a miscalculation of insulin dose.

The most common circumstance for the most recent HE was while doing household jobs or shopping (22.1%), followed by relaxing (watching TV, reading; 21.5%), and just prior to eating (9.9%), during leisure/social activities (9.3%) and while sleeping (9.3%). Only 8.1% of the most recent HEs were while at work, though much of the sample was not employed. Of those employed, at work was the most commonly reported location (23.0%).

Most of the most recent HEs described were self-managed (85.2%), but 10.1% required assistance from another non-medical person and 4.7% needed medical assistance. The most typical response was to eat or drink something (76.4%) and/or take glucose tablets (21.7%), or discontinue physical activity (9.4%). Some patients began checking their blood glucose more frequently (16.8%) and (3.4%) had their doctor change their treatment regimen as a result of the most recent HE.

### Outcome differences associated with HE

Comparisons between those with ≥1 HE in the prior four weeks and those without an event during the same period of time revealed numerous differences (Table 3). Of primary interest, among the portion of the sample providing a value for HbA1c at baseline or the first follow-up survey, those with ≥1 HE in the preceding four week had significantly higher A1c values than those who did not (p < 0.01). The EQ-5D health state index score was lower (worse) among those experiencing ≥1 HE in 4 weeks prior to the first survey relative to those without (p < 0.0001). Differences in work impairment between the two groups were also apparent. Employed respondents who experienced ≥1 HE in the month leading to the baseline survey reported approximately double the work impairment in the prior seven days experienced by those employed respondents without ≥1 HE, an absolute difference of 18.7%. Impairment to non-work activities during the same period was also higher among those with ≥1 HE, equating to a mean absolute difference of 12.5%. Those who had experienced ≥1 HE in the past week attributed considerable impairment to the event, 24.6% overall work impairment among the employed, and 44% impairment in non-work activities (not presented).

**Table 3:** Bivariate health outcome differences between patients reporting hypoglycaemia and patients not experiencing hypoglycaemia

	**Hypoglycaemia (n = 365)**		**No hypoglycaemia (n = 964)**		
	**n/mean**	**%/SD**	**n/mean**	**%/SD**	**p-value**
HbA1c (n =139 & 254)	7.51	1.60	7.11	1.39	0.0095
EQ-5D Index (n = 360 & 953)	0.56	0.39	0.69	0.34	<0.0001
Work and activity impairment					
Overall work impairment (n = 141 & 270)	35.3%	35.8%	16.6%	25.6%	<0.0001
Activity impairment	44.5%	32.8%	32.0%	31.7%	<0.0001
Treatment satisfaction	0.67	0.17	0.75	0.18	<0.0001
Adherence to treatment					
Oral medications (n = 316 & 911)	0.81	1.01	0.69	0.94	0.0573
Insulin (n = 131 & 170)	0.95	1.25	0.60	0.96	0.0076
Diabetes-related healthcare resource use					
4-week PCP visits	0.73	1.05	0.38	0.66	<0.0001
4-week total visits	1.82	2.50	0.92	1.51	<0.0001
4-week hospitalizations	0.16	0.52	0.06	0.31	0.0006
Hypoglycaemia fear					
Worry	15.60	12.51	6.53	9.24	<0.0001
Behaviour	17.81	15.39	6.58	10.95	<0.0001
Total	33.40	25.46	13.11	18.46	<0.0001

Note: When fewer than the full sample are included in a comparison, sample sizes are indicated in parentheses for those experiencing hypoglycaemia and not experiencing hypoglycaemia in the four weeks prior to the survey, respectively. Lower HbA1c is associated with better outcomes and fewer complications. Higher EQ-5D scores indicate more preferred health status, with 1.0 equivalent to perfect health. Work impairment and activity impairment ranged from 0 to 100%, with lower numbers indicating less impairment. Treatment satisfaction ranges from 0 to 1.0, with higher numbers indicating grater satisfaction. Adherence to treatment ranges from 0 to 4, with higher numbers indicating less adherence. Hypoglycemia fear ranges from 0 to 72 for Worry, 0 to 60 for behaviour, and 0 to 132 for the total score, with higher scores indicating more fear of hypoglycaemia.

Satisfaction with medication and adherence to medications were also different based on the experience of ≥1 HE in the prior four weeks. Medication satisfaction was lower among those who experienced ≥1 HE in the 4 weeks prior to survey (.08 on a 0–1.0 scale; p < 0.0001). Adherence to oral medication was only marginally lower (p = .06) in those with at ≥1 HE in the prior four weeks, and adherence to insulin was significantly lower among insulin users experiencing ≥1 HE during that time relative to insulin users who did not (p < 0.0001).

Experience of ≥1 HE in the past month was associated with increased healthcare resource use relative to those who did not. Patients who experienced ≥1 HE visited the physician (who primarily manages their T2D) almost twice as often as those who did not. A similar increase was seen in the number of total diabetes-related healthcare visits. There were relatively few diabetes-related hospitalizations reported among either group, but the average among those reporting ≥1 HE was more than twice that of those who did not report any HEs.

Fear of hypoglycaemia was significantly higher among those experiencing ≥1 HE for the total score as well as both subscales (p < 0.0001). Respondents with ≥1 HE reported performing a range of specific glucose-raising behaviours on the HFS-II (Table 4).

**Table 4:** Blood-glucose increasing behaviours in HFS-II used within last 4 weeks by experience of hypoglycaemia during that period

	**Hypoglycaemia (n = 365)**		**No hypoglycaemia (n = 964)**		
	**n**	**%**	**n**	**%**	**p-value**
Ate large snacks	283	77.5%	497	51.6%	<0.0001
Kept glucose above 150	225	61.6%	352	36.5%	<0.0001
Kept glucose higher than normal in social situations	199	54.5%	189	19.6%	<0.0001
Kept glucose higher than normal in important activities	205	56.2%	197	20.4%	<0.0001
Reduced insulin dose (n = 131 & 170)	91	69.5%	88	51.8%	0.0021

Baseline data of those completing all surveys was explored for differences between those reporting ≥1 HE during the course of the study (n = 83) and those who did not experience HE (n = 72). This analysis included only a small and self-selected subsample of the baseline survey respondents, but is included here to facilitate hypothesis generation for future research (Table 5). The mean difference in HbA1c averaged across each wave (0.3%) was consistent with the baseline difference, but not statistically significant. The mean EQ-5D index score for those with ≥1 HE during the study was .09 points worse that of those who completed the study without experiencing any HE, a difference which approached statistical significance (p =0.07) and would exceed the 0.07 threshold for minimally important difference [32]. There was greater activity impairment among the group that experienced ≥1 HE, but no significant difference in work impairment. The 8% decrement in satisfaction with diabetes medication observed in the baseline comparison was observed in the longitudinal sample. Greater use of healthcare resources was observed among those reporting ≥1 HE, although this difference was limited to the total number of provider visits. The most significant differences in the longitudinal subsample were of average scores on the HFS-II, all of which remained significantly worse (all p <0.001), with total scores 15 points higher among those with ≥1 HE.

**Table 5:** Average outcomes among those completing all study surveys

	**Hypoglycaemia (n = 83)**		**No hypoglycemia (n = 72)**		
	**Mean**	**SD**	**Mean**	**SD**	**p-value**
HbA1c (LOCF; n = 53 & 37)	7.35%	1.68%	7.05%	1.83%	0.4389
Health-related quality of life					
EQ-5D index	0.62	0.35	0.71	0.29	0.0669
Work and activity impairment					
Overall work impairment	19.6%	21.9%	10.8%	22.5%	0.1620
Activity impairment	41.2%	29.2%	28.9%	29.4%	0.0102
Treatment satisfaction	0.72	0.16	0.80	0.15	0.0011
Diabetes-related healthcare resource use					
4-week diabetes PCP visits	0.43	0.48	0.31	0.41	0.0948
4-week total diabetes provider visits	1.07	1.24	0.69	0.88	0.0286
4-week total hospitalizations	0.05	0.15	0.06	0.24	0.9661
Hypoglycaemia fear					
Worry	11.59	9.72	4.45	5.31	<0.0001
Behaviour	11.61	11.74	3.26	5.72	<0.0001
Total	23.19	20.25	7.71	9.47	<0.0001

Note: LOCF, last observation carried forward.

## Discussion

The current study found that HEs are common in patients with T2D in the UK, with 23% of those using oral medication (without insulin) experiencing a HE during a four week period, while the rate among insulin users was nearly double (44%). Those who experienced ≥1 HE in the prior week attributed substantial impairment to both work and activities of daily living. Comparisons of those who experienced ≥1 HE in the four weeks prior to the survey also had worse outcomes, including health status and treatment satisfaction, more diabetes-specific healthcare resource use, greater impairment of non-work activities, and more fear of hypoglycaemia, including a greater likelihood of reporting behaviours that would raise their blood glucose levels. Insulin users who reported ≥1 HE also reported less adherence to their insulin than users who were free of HEs; the relationship between HEs and adherence was not observed in oral medications, perhaps because patients understand the potential for insulin to cause hypoglycaemia, and perhaps due to the injectable mode of administration, which is more aversive than swallowing a pill and also enables dose reduction.

Although the 0.3% difference in HbA1c is modest relative to the effect of antihyperglycaemic medications [33], it indicates that HEs may be predictive of higher HbA1c when linked to the results indicating HEs to be associated with behaviour likely to increase glucose levels.

The other differences between the groups are larger in magnitude. The mean difference in EQ-5D index of 0.13 points represents an effect of d = 0.36, equal or larger to some guidelines for a clinically meaningful effect [34]. This difference was equal or slightly larger than the 0.08-0.10 difference reported in similar studies in other geographies [20,22]. This finding may be particularly important, given the established predictive relationship between EQ-5D scores and both complications and mortality in diabetes [33,35]. The magnitude of some of the other disparities are larger still; applying the standard Cohen’s d effect size guidelines [36], the 0.08 point difference in treatment satisfaction would be interpreted as a moderate (d = 0.44) effect, while the differences in hypoglycaemia fear are large, ranging from d = 0.82 for worry to d = 0.90 for the total score.

One finding of this study that is at odds with previous research is the relationship between HEs and HbA1c. Though few studies have investigated the connection between HEs and glycaemic control, an Asian study measuring HbA1c from blood draw found lower HbA1c among those with hypoglycaemia [24]. It is unclear why the findings are different, but it could be that the relationship between hypoglycaemia and glycaemic control cannot be clearly ascertained from cross-sectional analyses, and there are reasons to believe that both relationships are true under certain circumstances. Indeed, those who maintain low blood glucose are, in a sense, closer to hypoglycaemia than those whose blood glucose is consistently high, and previous research has demonstrated that individuals with lower glycosylated hemoglobin levels are indeed at greater risk of HEs [7,9,37,38]. However, those who experience HEs may adjust their regimen in an attempt to prevent subsequent HEs, potentially raising their HbA1c in the process [39,40]. It could also be that relationship between glycaemic control and HEs differs according to region due to cultural or lifestyle factors.

Another interesting finding was the location of the HEs, which were most commonly reported as happening in the context of household jobs or relaxing rather than during physical activity such as exercise or playing sports which might be expected to result in low blood glucose levels. Unfortunately, this is likely due to lack of physical activity among the sample; less than half of the sample reported one or more days in the previous month during which they exercised for 20 minutes or more, and items asking about the context of the HE referred only to the most recent episode to maximize the accuracy of the recall. If individuals exercise infrequently, they have limited opportunities to have a HE during exercise.

There are some limitations of the study that should be acknowledged. An important limitation shared by this and other real-world studies on HEs is the reliance on a sample other than a population-based probability sample; in this case a sample from an opt-in survey panel. Therefore, this sample may under- or overestimate the prevalence of HEs in the UK T2D population. This also makes it difficult to determine the extent to which the other results of the present study generalize to the broader UK T2D population, though the sample appears representative on several important characteristics. The use of the online survey panel also limited follow-up reminders to email which, along with the limited response window, likely contributed to the relatively low response rate for the follow-up waves. Another limitation is the lack of HbA1c results for the majority of the sample, which limited statistical power and raises the concern that this portion of the sample may be different in important ways from the participants who were unable to provide an HbA1c value. Finally, the analyses here are primarily cross-sectional and correlational in nature. Comparisons were not adjusted for potential differences between the groups in terms of demographic, disease, or treatment characteristics, and differences between the groups other than the presence of HEs may explain the differences in outcomes.

While not a limitation per se, an important point to consider is that HE status relied on self-report. Patient characterizations of their most recent HE revealed that many did not test blood glucose during their episode, and a few claimed to have normal blood glucose levels during their HE, raising the question of whether all those in the HE group experienced a HE as defined by the American Diabetes Association criteria [41]. From the standpoint of behaviour, this may not be important; if the perception that one is experiencing HEs result in less effective disease management, then whether the blood glucose level is actually low is of little importance. However, if the response to the HE is to switch the patient to a medication that has a lower risk of HEs, such a switch may not have the intended result because what the patient perceived as hypoglycaemia was something else.

Despite these caveats, the current study makes some meaningful contributions to our understanding of HEs among UK patients with T2D, estimating the four-week prevalence of HEs among those on antihyperglycaemic medication, characterizing the events, and documenting the differences between patients with and without recent HEs. Unlike previous some previous studies that included patients with type 1 diabetes, the current study focused entirely on T2D. The sample was considerably larger than the UK T2D samples in previous studies, and the survey included a broader array of patient-reported outcomes. The present study also confirms findings from other studies in other geographies regarding the relationship between HEs and impairment to work and non-work activities, satisfaction with medication, healthcare utilization, and fear of hypoglycaemia. The findings underscore the importance of considering risk of HEs when planning a patient’s treatment regimen, ensuring patients are educated about HEs, and encouraging regular testing of blood glucose.

## Conclusion

HEs are unpleasant, relatively common among those being treated for T2D, and are feared by patients even if they have not had recent experience of one. Those who report recently experiencing ≥1 HE have worse health outcomes than those who do not. In addition to impaired outcomes such as health status and treatment satisfaction, experiencing ≥1 HE is associated with worry and performing behaviours designed to increase blood glucose, with the likely eventual result being impaired glucose control, an increased risk of complications, worsening health outcomes for the patient, and increased burden on the healthcare system. Future research should assess whether interventions aimed at reducing HEs and fear of HEs also improve outcomes.

## Competing interests

AZ, BC, BDM, and MR are employees of Eli Lilly & Company. JV is an employee of Kantar Health. The study and preparation of the manuscript was conducted by Kantar Health with funding from Eli Lilly & Company.

## Authors’ contributions

BDM conceived of the study, contributed to the study design, and revised the manuscript for important intellectual content. JV conducted the statistical analysis and drafted the manuscript. AZ contributed to the design of the study and statistical analysis plan. MR and BC contributed to the study design and revised the draft manuscript for important intellectual content. All authors have read and approved the final manuscript.

## Pre-publication history

The pre-publication history for this paper can be accessed here:

http://www.biomedcentral.com/1472-6823/13/59/prepub

## References

[B1] Patel A MacMahon S Chalmers J Neal B Billot L Woodward M Marre M Cooper M Glasziou P Grobbee D Intensive blood glucose control and vascular outcomes in patients with type 2 diabetes N Engl J Med 2008 13 2560 2572 18539916 10.1056/NEJMoa0802987

[B2] The effect of intensive treatment of diabetes on the development and progression of long-term complications in insulin-dependent diabetes mellitus. The Diabetes Control and Complications Trial Research Group N Engl J Med 1993 13 977 986 8366922 10.1056/NEJM199309303291401

[B3] Durante AJ Selwyn PA O'Connor PG Risk factors for and knowledge of Mycobacterium tuberculosis infection among drug users in substance abuse treatment Addiction 1998 13 1393 1401 10.1046/j.1360-0443.1998.939139310.x 9926545

[B4] Gaede P Vedel P Larsen N Jensen GV Parving HH Pedersen O Multifactorial intervention and cardiovascular disease in patients with type 2 diabetes N Engl J Med 2003 13 383 393 10.1056/NEJMoa021778 12556541

[B5] Shichiri M Kishikawa H Ohkubo Y Wake N Long-term results of the Kumamoto Study on optimal diabetes control in type 2 diabetic patients Diabetes Care 2000 13 Suppl 2 B21 B29 10860187

[B6] Ohkubo Y Kishikawa H Araki E Miyata T Isami S Motoyoshi S Kojima Y Furuyoshi N Shichiri M Intensive insulin therapy prevents the progression of diabetic microvascular complications in Japanese patients with non-insulin-dependent diabetes mellitus: a randomized prospective 6-year study Diabetes Res Clin Pract 1995 13 103 117 10.1016/0168-8227(95)01064-K 7587918

[B7] Gerstein HC Miller ME Byington RP Goff DC Jr Bigger JT Buse JB Cushman WC Genuth S Ismail-Beigi F Grimm RH Jr Effects of intensive glucose lowering in type 2 diabetes N Engl J Med 2008 13 2545 2559 18539917 10.1056/NEJMoa0802743 PMC4551392

[B8] Kelly TN Bazzano LA Fonseca VA Thethi TK Reynolds K He J Systematic review: glucose control and cardiovascular disease in type 2 diabetes Ann Intern Med 2009 13 394 10.7326/0003-4819-151-6-200909150-00137 19620144 PMC3058557

[B9] Duckworth W Abraira C Moritz T Reda D Emanuele N Reaven PD Zieve FJ Marks J Davis SN Hayward R Glucose control and vascular complications in veterans with type 2 diabetes N Engl J Med 2009 13 129 139 10.1056/NEJMoa0808431 19092145

[B10] Currie CJ Peters JR Tynan A Evans M Heine RJ Bracco OL Zagar T Poole CD Survival as a function of HbA1c in people with type 2 diabetes: a retrospective cohort study Lancet 2010 13 481 489 10.1016/S0140-6736(09)61969-3 20110121

[B11] Harris MI Health care and health status and outcomes for patients with type 2 diabetes Diabetes Care 2000 13 754 10.2337/diacare.23.6.754 10840991

[B12] Saaddine JB Engelgau MM Beckles GL Gregg EW Thompson TJ Narayan K A diabetes report card for the United States: quality of care in the 1990s Ann Intern Med 2002 13 565 10.7326/0003-4819-136-8-200204160-00005 11955024

[B13] Blak BT Smith HT Hards M Maguire A Gimeno V A retrospective database study of insulin initiation in patients with Type 2 diabetes in UK primary care Diabet Med 2012 13 e191 e198 10.1111/j.1464-5491.2012.03694.x 22507537

[B14] Holman RR Farmer AJ Davies MJ Levy JC Darbyshire JL Keenan JF Paul SK Three-year efficacy of complex insulin regimens in type 2 diabetes N Engl J Med 2009 13 1736 1747 10.1056/NEJMoa0905479 19850703

[B15] Alvarez Guisasola F Tofe Povedano S Krishnarajah G Lyu R Mavros P Yin D Hypoglycaemic symptoms, treatment satisfaction, adherence and their associations with glycaemic goal in patients with type 2 diabetes mellitus: findings from the Real-Life Effectiveness and Care Patterns of Diabetes Management (RECAP-DM) Study Diabetes Obes Metab 2008 13 Suppl 1 25 32 18435671 10.1111/j.1463-1326.2008.00882.x

[B16] Ross SA Tildesley HD Ashkenas J Barriers to effective insulin treatment: the persistence of poor glycemic control in type 2 diabetes Curr Med Res Opin 2011 13 Suppl 3 13 20 21942467 10.1185/03007995.2011.621416

[B17] Leiter LA Yale J Chiasson J Harris SB Kleinstiver P Sauriol L Assessment of the impact of fear of hypoglycemic episodes on glycemic and hypoglycemia management Can J Diabetes 2005 13 186 192

[B18] Wild D von Maltzahn R Brohan E Christensen T Clauson P Gonder-Frederick L A critical review of the literature on fear of hypoglycemia in diabetes: Implications for diabetes management and patient education Patient Educ Couns 2007 13 10 15 10.1016/j.pec.2007.05.003 17582726

[B19] Peyrot M Barnett A Meneghini L Schumm‒Draeger PM Insulin adherence behaviours and barriers in the multinational Global Attitudes of Patients and Physicians in Insulin Therapy study Diabet Med 2012 13 682 689 10.1111/j.1464-5491.2012.03605.x 22313123 PMC3433794

[B20] Williams SA Pollack MF DiBonaventura M Effects of hypoglycemia on health-related quality of life, treatment satisfaction and healthcare resource utilization in patients with type 2 diabetes mellitus Diabetes Res Clin Pract 2011 13 363 370 10.1016/j.diabres.2010.12.027 21251725

[B21] Brod M Skovlund SE Wittrup-Jensen KU Measuring the impact of diabetes through patient report of treatment satisfaction, productivity and symptom experience Qual Life Res 2006 13 481 491 10.1007/s11136-005-1624-6 16547787

[B22] Marrett E Radican L Davies MJ Zhang Q Assessment of severity and frequency of self-reported hypoglycemia on quality of life in patients with type 2 diabetes treated with oral antihyperglycemic agents: a survey study BMC Res Notes 2011 13 251 10.1186/1756-0500-4-251 21777428 PMC3148563

[B23] Williams SA Shi L Brenneman SK Johnson JC Wegner JC Fonseca V The burden of hypoglycemia on healthcare utilization, costs, and quality of life among type 2 diabetes mellitus patients J Diabetes Complications 2012 13 399 406 10.1016/j.jdiacomp.2012.05.002 22699113

[B24] Sheu WH Ji LN Nitiyanant W Baik SH Yin D Mavros P Chan SP Hypoglycemia is associated with increased worry and lower quality of life among patients with type 2 diabetes treated with oral antihyperglycemic agents in the Asia-Pacific region Diabetes Res Clin Pract 2012 13 141 148 10.1016/j.diabres.2011.12.027 22265956

[B25] Charlson M Szatrowski TP Peterson J Gold J Validation of a combined comorbidity index J Clin Epidemiol 1994 13 1245 1251 10.1016/0895-4356(94)90129-5 7722560

[B26] Rabin R Charro F EQ-5D: a measure of health status from the EuroQol Group Ann Med 2001 13 337 343 10.3109/07853890109002087 11491192

[B27] Reilly MC Zbrozek AS Dukes EM The validity and reproducibility of a work productivity and activity impairment instrument Pharmacoeconomics 1993 13 353 10.2165/00019053-199304050-00006 10146874

[B28] Anderson RT Girman CJ Pawaskar MD Camacho FT Calles J Kelly WS DeMuro C Balkrishnan R Diabetes Medication Satisfaction Tool: a focus on treatment regimens Diabetes Care 2009 13 51 53 10.2337/dc08-0856 18931098 PMC2606829

[B29] Morisky DE Green LW Levine DM Concurrent and predictive validity of a self-reported measure of medication adherence Med Care 1986 13 67 74 10.1097/00005650-198601000-00007 3945130

[B30] Morisky DE Malotte CK Choi P Davidson P Rigler S Sugland B Langer M A patient education program to improve adherence rates with antituberculosis drug regimens Health Educ Behav 1990 13 253 10.1177/109019819001700303 2228629

[B31] Cox DJ Irvine A Gonder-Frederick L Nowacek G Butterfield J Fear of hypoglycemia: quantification, validation, and utilization Diabetes Care 1987 13 617 621 10.2337/diacare.10.5.617 3677982

[B32] Walters SJ Brazier JE Comparison of the minimally important difference for two health state utility measures: EQ-5D and SF-6D Qual Life Res 2005 13 1523 1532 10.1007/s11136-004-7713-0 16110932

[B33] McEwen LN Kim C Haan MN Ghosh D Lantz PM Thompson TJ Herman WH Are health-related quality-of-life and self-rated health associated with mortality? Insights from Translating Research Into Action for Diabetes (TRIAD) Prim Care Diabetes 2009 13 37 42 10.1016/j.pcd.2009.01.001 19269911 PMC4138696

[B34] Norman GR Sloan JA Wyrwich KW The truly remarkable universality of half a standard deviation: confirmation through another look Expert Rev Pharmacoecon Outcomes Res 2004 13 581 585 10.1586/14737167.4.5.581 19807551

[B35] Clarke PM Hayes AJ Glasziou PG Scott R Simes J Keech AC Using the EQ-5D index score as a predictor of outcomes in patients with type 2 diabetes Med Care 2009 13 61 68 10.1097/MLR.0b013e3181844855 19106732

[B36] Cohen J Statistical power analysis for the behavioral sciences 1988 2 Hillsdale, NJ: Lawrence Erlbaum

[B37] Lipska KJ Warton EM Huang ES Moffet HH Inzucchi SE Krumholz HM Karter AJ HbA1c and risk of severe hypoglycemia in type 2 diabetes: the diabetes and aging study Diabetes Care 2013 13 3535 3542 10.2337/dc13-0610 23900589 PMC3816866

[B38] Mancia G Grassi G Protection of patients with diabetes, with or without hypertension: implications of ADVANCE for clinical practice J Hypertens Suppl 2009 13 S19 S23 19483504 10.1097/01.hjh.0000354420.23557.e2

[B39] Ritholtz MD Jacobson AM Living with hypoglycemia J Gen Intern Med 1998 13 799 804 10.1046/j.1525-1497.1998.00243.x 9844077 PMC1497041

[B40] Richmond J Effects of hypoglycaemia: patient’s perceptions and experiences Br J Nurs 1996 13 1054 1059 8918765 10.12968/bjon.1996.5.17.1054

[B41] Seaquist ER Anderson J Childs B Cryer P Dagogo-Jack S Fish L Heller SR Rodriguez H Rosenzweig J Vigersky R Hypoglycemia and diabetes: a report of a workgroup of the American Diabetes Association and The Endocrine Society Diabetes Care 2013 13 1384 1395 10.2337/dc12-2480 23589542 PMC3631867

